# Cost effectiveness of a multi-component school-based physical activity intervention targeting adolescents: the ‘Physical Activity 4 Everyone’ cluster randomized trial

**DOI:** 10.1186/s12966-016-0418-2

**Published:** 2016-08-22

**Authors:** Rachel Sutherland, Penny Reeves, Elizabeth Campbell, David R. Lubans, Philip J. Morgan, Nicole Nathan, Luke Wolfenden, Anthony D. Okely, Karen Gillham, Lynda Davies, John Wiggers

**Affiliations:** 1 Hunter New England Population Health, Locked Bag 10, Wallsend, NSW Australia 2287; 2 School of Medicine and Public Health, University of Newcastle, Newcastle, 2308 Australia; 3 Hunter Medical Research Institute, Newcastle, NSW Australia 2305; 4 Priority Research Centre in Physical Activity and Nutrition, School of Education, University of Newcastle, Newcastle, Australia 2308; 5 Early Start Research Institute and School of Education, University of Wollongong, Wollongong, NSW Australia 2500; 6 Illawarra Health and Medical Research Institute, Wollongong, NSW Australia 2522

**Keywords:** Physical activity, Adolescents, School, Randomized controlled trial, Low income, Disadvantaged, Cost effectiveness, Economic

## Abstract

**Background:**

Few school-based interventions have been successful in reducing physical activity decline and preventing overweight and obesity in adolescent populations. As a result, few cost effectiveness analyses have been reported. The aim of this paper is to report the cost and cost effectiveness of the Physical Activity 4 Everyone (PA4E1) intervention which was a multi-component intervention implemented in secondary schools located in low-income communities. Cost effectiveness was assessed using both the physical activity and weight status trial outcomes.

**Methods:**

*Intervention and Study Design:* The PA4E1 cluster randomised controlled trial was implemented in 10 Australian secondary schools (5 intervention: 5 control) and consisted of intervention schools receiving seven physical activity promotion strategies and six additional strategies that supported school implementation of the intervention components. Costs associated with physical activity strategies, and intervention implementation strategies within the five intervention schools were estimated and compared to the costs of usual physical activity practices of schools in the control group. The total cost of implementing the intervention was estimated from a societal perspective, based on the number of enrolled students in the target grade at the start of the intervention (Grade 7, *n* = 837).

*Economic Outcomes:* The economic analysis outcomes were cost and incremental cost effectiveness ratios for the following: minutes of moderate-to-vigorous physical activity (MVPA) per day gained, MET hours gained per person/day; Body Mass Index (BMI) unit avoided; and 10 % reduction in BMI z-score.

**Results:**

The intervention cost AUD $329,952 over 24 months, or AUD$394 per student in the intervention group. This resulted in a cost effectiveness ratio of AUD$56 ($35–$147) per additional minute of MVPA, AUD$1 ($0.6–$2.7) per MET hour gained per person per day, AUD$1408 ($788–$6,570) per BMI unit avoided, and AUD$563 ($282–$3,942) per 10 % reduction in BMI z-score.

**Conclusion:**

PA4E1 is a cost effective intervention for increasing the physical activity levels and reducing unhealthy weight gain in adolescence, a period in which physical activity typically declines. Additional modelling could explore the potential economic impact of the intervention on morbidity and mortality.

**Trial registration:**

Australian New Zealand Clinical Trials Registry ACTRN12612000382875.

## Background

Regular physical activity has well established positive benefits for both physical and mental health [1], yet physical activity levels are known to decline throughout adolescence [2] with only 20 % of youth currently undertake sufficient daily physical activity to obtain these health benefits [3]. Physical inactivity is considered to directly contribute to 1.5 %–3.0 % of global health care costs [4], including direct and indirect health care costs [5]. The large proportion of low-active adolescents, coupled with the global concern regarding overweight and obesity, make population-based interventions focused on physical activity promotion and obesity prevention in this population sub-group a public health priority [6–8]. As both physical inactivity and overweight and obesity are more prevalent in adolescents from disadvantaged backgrounds, strategies targeting this population are particularly warranted [9, 10].

School-based physical activity and lifestyle interventions show promise in addressing both physical inactivity and overweight and obesity [6–8, 11–13]. Schools provide almost universal access to children and adolescents, including those from disadvantaged backgrounds [14]. In addition, schools have the policies, resources, and teaching staff to adopt programs into usual school practice that are likely to impact on both physical activity and weight status [15]. Despite this, successful interventions targeting adolescents are limited in number, particularly interventions that target adolescents from disadvantaged backgrounds [7, 12, 16–20]. A recent systematic review reported only 14 of the 44 included school-based physical activity intervention trials targeted adolescents, of which only four resulted in significant physical activity intervention effects [7]. Only two of the adolescent trials focused on disadvantaged adolescents, with one reporting significant intervention effects on physical activity [7]. Additionally, a recent review of childhood and adolescent obesity prevention reported multi-component school physical activity interventions have resulted in only modest reductions in BMI (−0.13 kg/m [2], 95 % CI −0.22 to −0.04) [21]. However, the review reported results for both children and adolescents combined, with the impact specifically on adolescents unknown. Systematic reviews of interventions that aim to prevent obesity have demonstrated smaller effects in adolescent populations in comparison to younger children [22].

In order for policy makers to allocate scarce health resources, economic evaluations of effective programs, ideally based on outcomes of randomised controlled trials, are needed [23]. Cost-effectiveness analysis (CEA) aims to evaluate questions around the benefits of interventions relative to their cost in order to inform funding decisions and health care policy [24] CEA is used to determine technical efficiency. That is, the production of health benefit for the least cost. No single threshold exists for determining the acceptability of a CE ratio. Rather, a variety of considerations, including the prosperity of a nation or health system, as well as the incremental value delivered by an intervention, influence funding decisions. Despite the valuable contribution of CEA, very few studies have evaluated school-based physical activity interventions from a cost effectiveness perspective [25–31]. Even fewer studies have targeted adolescents and none have focussed on disadvantaged adolescents. Two recent systematic reviews of physical activity interventions reporting cost-effectiveness included school-based interventions, but neither separated the effects for elementary and secondary school-focussed interventions [27, 31]. The reported cost effectiveness of interventions included in the review by Wu and colleagues (16 school-based trials, four in adolescents) was based on costs obtained either directly from published cost analyses or imputed by the review authors [31]. The second review by Laine and colleagues included school-based interventions from the Wu review [31], as well as modelled cost-effectiveness studies [27] (total of six school based trials, three in adolescents). While these reviews have limitations such as few of the studies assessing physical activity using objective measures [32], use of imputed cost estimates rather than actual costs and variability in study design (with rigorous well designed RCT’s tending to show smaller physical activity effectiveness and higher cost-effectiveness ratios) [27] [31], both reviews conclude that school-based physical activity interventions are cost effective compared to other population based interventions in terms of physical activity outcomes [27, 31].

From an obesity prevention perspective, the Australian ACE Obesity prevention study conducted in 2003 used modelling techniques to review a portfolio of interventions targeting the prevention of childhood obesity [33]. Five of the thirteen population level interventions were school-based. The review concluded that multi-strategic school-based interventions were cost effective (modelled to cost less than $50,000 AUD per DALY) and estimated at $211–$473 per student [33]. However the strength of the evidence was often limited, weak or inconclusive with only seven of the 13 interventions included in the study being based on evidence of effect gained from randomized controlled trials [33]. A further systematic review of eight childhood obesity primary prevention trials (including three school-based trials all targeting elementary aged children) reported school-based interventions were cost effective using a variety of cost effectiveness measures [34]. The authors concluded that limited comparison between studies could be made due to the heterogeneity of outcome measures across the studies, low quality of included studies and the use of model-based studies to obtain an outcome rather than trial outcome measures. Given the limitations of existing data there is an increasing demand for additional data on cost and cost effectiveness of school based intervention for both physical activity and adiposity outcomes.

The Physical Activity 4 Everyone (PA4E1) trial involved a 24-month multicomponent school-based intervention implemented in secondary schools located in disadvantaged communities [35]. The trial aimed to determine the effectiveness of the intervention in reducing the decline in physical activity among adolescents. The trial was one of a very limited number of school-based physical activity interventions that has demonstrated an increase in objectively measured physical activity coupled with a reduction in weight gain [36–38], and the first study in adolescents [39, 40]. At both 12 [35] and 24-months [39], the study reported improvements in daily moderate-to-vigorous physical activity (MVPA) together with a positive effect for weight and body mass index (BMI) in favour of the intervention group [35, 39, 40]. In addition, a significant intervention effect was also observed for BMI Z-score at 24 months [40]. Due to the limited literature outlining the cost effectiveness of school-based interventions that can impact on both physical activity and weight status in adolescents, the aim of this study was to assess the costs of the PA4E1 intervention, and the cost effectiveness of the intervention considering both physical activity and weight status trial outcomes.

## Methods

### Intervention trial design, setting and sample

A cluster randomized trial was conducted involving randomly selected secondary schools (five intervention and five control schools) in socio-economically disadvantaged communities in New South Wales (NSW), Australia. Outcome assessments were conducted with a cohort of students at baseline (when students were in Grade 7), 12-month (mid-intervention) and 24-month post-randomisation follow-up. Details of the study methods have been reported elsewhere [16], along with the intervention effects at12-months [35] and 24-months [39, 40].

The trial was registered with the Australian New Zealand Clinical Trials Registry (ACTRN1261200038287) and approved by the Hunter New England Area Human Research Ethics Committee (11/03/16/4.0) and the University of Newcastle Human Research Ethics Committee (H-2011-0210). The study adhered to the Consolidated Standards of Reporting Trials (CONSORT) guidelines (http://www.consort-statement.org), and the Consolidated Health Economic Evaluation Reporting Standards (CHEERS) Statement (http://www.equator-network.org/reporting-guidelines/cheers/) [41].

### Economic study and setting

A trial-based retrospective economic evaluation of a multi-component school-based physical activity outcome (PA4E1) versus usual school physical activity practice was conducted from a societal perspective. The outcomes for the economic analysis were the cost and incremental cost effectiveness ratios per: minute of MVPA per day gained; MET hour gained per person/ day; BMI unit avoided; and 10 % reduction in BMI z-score.

### PA4E1 intervention

**Fig. 1:**
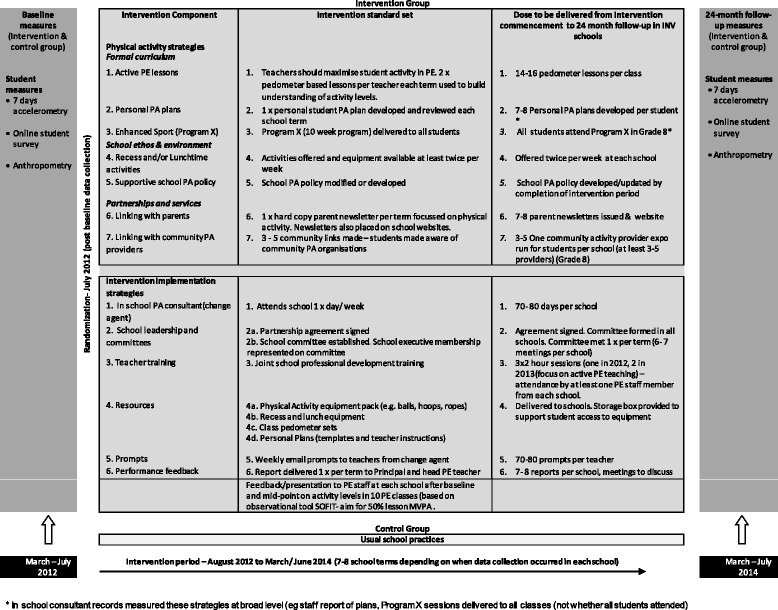
Intervention overview – physical activity and intervention implementation strategies

The intervention implemented in secondary schools based in disadvantaged communities located in NSW, Australia, was delivered to all students who commenced Grade 7 in 2012, through incorporating the intervention as part of usual school business. The intervention was implemented over 7–8 school terms (average 24 months) and consisted of embedding seven physical activity strategies across the domains of the Health Promoting Schools Framework [42] into the school community. The seven physical activity strategies included: more active physical education (PE) lessons; development of personal physical activity plans; delivery of a 10 week enhanced school sport program (Program X [43, 44]); conducting supervised recess and/or lunch physical activity opportunities; supportive school physical activity policy; and linking with the community and linking with parents (Fig. 1. Intervention overview – physical activity and intervention implementation strategies [39]). In addition to the physical activity strategies, six evidenced-based implementation support strategies were delivered [45–49]: an in-school physical activity consultant, executive support, teacher training, resources, prompts and monitoring reports (Fig. 1). Schools allocated to the control group participated in the measurement components of the trial only and delivered physical activity teaching and promotion practices according to the PE curriculum and school-based initiatives. Intervention materials were provided to control schools following the 24-month assessments.

### Measurement of trial outcomes

#### Physical activity

Accelerometer data were used to derive the physical activity outcome measure, duration (minutes) of MVPA per day. Accelerometer non-wear time was defined as 30 min of consecutive zeros [50]. Counts were collected in 15 s epochs and counts per minute calculated by dividing the total accelerometer counts by the minutes of wear time. The Evenson cut-points were used to categorise the intensity of physical activity (moderate or vigorous) [51]. Mins per day of MVPA were calculated for students who wore accelerometers for ≥ 600 min on ≥ 3 days [52].

The conversion of minutes of MVPA per day to MET hours gained per person/ day was undertaken to aid the comparison with other cost effectiveness studies of physical activity interventions. A MET represents energy expended divided by resting energy expenditure [31, 53]. Determining MET hours gained accounts for the variety of physical activity measures in use and takes into account a range of parameters including intensity, duration and frequency of physical activity [31].

To determine MET hour gained per person/day, the difference in daily minutes of MVPA between the intervention and the control groups was converted to MET hours gained, following the steps outlined by Wu et al., and replicated in a subsequent systematic review by Laine et al [27]. Using validated measures, moderate physical activity is assigned 3.0 to 6.0 METS, vigorous activity >6.0 METS and MVPA is assigned 4.5 METS [27]. This process of converting minutes of MVPA per day to MET hours gained involves multiplying mean minutes MVPA/ day by MET assigned, divided by 60 min.

#### Weight status

Anthropometric data were collected in duplicate by trained research assistants using the International Society for Advanced Kinathropometry (ISAK) procedures to measure student height and weight. [54] Students completed the measurements in light clothing without shoes. Weight was measured to the nearest 0.1 kg on a portable digital scale (Model no. UC-321PC, A&D Company Ltd, Tokyo Japan). Height was measured to the nearest 0.1 cm using a portable stadiometer (Model no. PE087, Mentone Educational Centre, Australia). Body mass index (BMI) was calculated (weight (kg) / height (m) [2]) and weight status determined using the International Obesity Taskforce definitions [55, 56].

#### Measurement of costs

The cost and incremental costs associated with the implementation of the physical activity intervention and intervention implementation strategies were calculated as those costs additional to the costs of usual physical activity practices of schools. The total cost of implementing the intervention was estimated from a societal perspective. Costs incurred for research and development were excluded in order to only capture the costs of replicating the intervention. Resource use categories included personnel costs, materials and printing. Personnel costs included opportunity costs for the delivery of strategies by school staff and community sport and fitness providers. All costs are reported in 2014 Australian dollars. All other resource use categories were valued using market rates. Potential effects on healthcare costs were not included.

#### Direct costs of the intervention

Project records relating to intervention delivery, including costs, were kept throughout the trial. For the physical activity strategies (Fig. 1), personnel costs included opportunity costs for delivery of strategies by school staff and community sport and fitness providers. Personnel costs for the implementation of strategies that occurred outside of PE and sport time were valued using the opportunity cost of forgone time. No opportunity costs were assumed for physical activity strategies 1–3 (Active PE, personal physical activity plans, enhanced sport) as such strategies were implemented by staff within school PE and sport time as part of usual school business. Opportunity costs were included for physical activity strategies 4, 5 and 7 (organised recess and/or lunch activities, policy, community links) as strategy 4 (organised recess and/or lunch activities) involved the provision of additional staffing of playground areas, strategy 5 (policy) required time for policy development/modification and sign off, and strategy 7 (community links) required time for school and community member involvement.

Costs incurred for the intervention implementation strategies (Fig. 1) included personnel costs, equipment and travel/venue/meal expenses. Personnel costs included in-school consultant salary, payment of consultants to deliver PE teacher training, teacher relief to allow PE teachers to attend training, and opportunity costs (forgone time) associated with implementation strategy 2 (school leadership and committee) as staff attended additional committee meetings about intervention implementation.

With respect to control schools, it was assumed that no additional costs were incurred in implementing their usual physical education practices.

Australian Bureau of Statistics average earnings data (May 2014) were used to impute labour costs for community sport and fitness personnel [57]. The Industrial Relations Commission of NSW 2014 Award data were used to impute labour costs for teaching personnel [58].

#### Statistical analysis

Cost effectiveness analysis was undertaken from a societal perspective and all analyses were carried out using Microsoft Excel software 2013. The analysis was conducted on an intention to treat basis, with the total program cost being calculated for all enrolled students in the target Grade across the five intervention schools at baseline given these students would have been exposed to the intervention (*n* = 837). Incremental cost effectiveness ratios (ICER) were calculated for each outcome measure and represent the additional expenditure required to deliver each additional unit of benefit.

For the physical activity outcome measures, the ICERs calculated were the cost per student per mean minute of MVPA gained and cost per student per MET minute gained. To present the intervention cost per minute of MVPA gain, the total cost per student was divided by the mean difference in change in MVPA minutes between intervention and control groups over 24 months, to provide a cost per student per minute of additional MVPA. The cost per person/day is then divided by the MET hours gained per day, resulting in a cost effectiveness ratio per MET hour gained [27, 31].

For the weight status outcomes, the ICERs were calculated to represent the expenditure per student per BMI unit avoided and cost per student per 0.1 unit (10 %) BMI z-score reduction. The total intervention cost per student was divided by mean difference in change in BMI and BMI z-score between groups over 24 months to provide a cost per BMI unit avoided and cost per 0.1 (10 %) reduction in BMI z-score [59].

The multicomponent intervention was delivered in its entirety to a cohort of students in Grade7 at the beginning of the intervention, followed through to Grade 9. Whilst the evaluation of the intervention occurred within the cohort of students and the cost effectiveness analysis has been conservatively calculated on the basis of the intervention benefiting only the cohort of students measured in the evaluation. Due to the nature of the intervention strategies (teacher training, school environment and broader school community links), it was likely the intervention had an impact on all students attending the school more broadly, not just on those students within the evaluation cohort. Univariate sensitivity analyses were undertaken to test plausible variation in the evaluation components as well as the impact of changing key design features of the intervention, including broader exposure and an associated estimate of benefit. Table 1 details the sensitivity tests that were modelled and provides justification for the assumptions made based on evaluations and empirical data from the PA4E1 trial: (i and ii) variation in the costs of specific intervention components (iii) variation in the magnitude of effect size using the upper and lower confidence interval limits; (iv) test assuming physical activity strategy 4 (recess and lunchtime activities) is extended to 10 % of students beyond the target grade, with a reduced effect on daily minutes of MVPA compared to students in the target grade; and (v) test assuming the benefits of physical activity strategy 1 (active PE), strategy 5 (physical activity policy) and implementation strategy 1 (change agent), 2 (executive support) and 3 (resources) are extended to all students (100 %) outside the target year (in Grades 7–10), with a reduced effect on daily minutes of MVPA compared to students in the target grade. Aggregated costs across schools meant it was not possible to capture the cost profiles of individual student participants, prohibiting uncertainty analysis.

**Table 1:** Sensitivity and scenario description: strategies and benefit

Test to be modelled	Detailed assumptions	Justification
Sensitivity analyses		
(i) Variation in the intervention cost	Higher estimate of the assumed opportunity cost of school staff participation in PA strategy (PAS) 4 & 5 and implementation support strategy (ISS) 1	Plausible variation in the cost
(ii) Variation in the intervention cost	Lower estimate of the assumed opportunity cost of school staff participation in PAS 4 & 5 and ISS 1	Plausible variation in the cost
(iii) Varying the magnitude of the effect size	Assumes benefit of the overall intervention varies between the calculated confidence interval of the effect size in daily minutes of MVPA	Plausible variation in the effect size
(iv) Extending the benefit of physical activity recess and lunchtime activities to students beyond the target year.	Assumes benefit of PAIS 4 is extended to 10 % of students beyond the target year, with a reduced effect on daily minutes of MVPA compared to students in the target year. Reduced effect estimate was based on the accelerometer data within the recess and lunchtime segment from the efficacy trial (unpublished).	It was likely these specific components of the intervention would impact students more broadly and not be isolated to those students within the evaluation cohort.
	The number of additional students that may benefit from whole of school recess and lunchtime activities was conservatively estimated based on 10 % of a multiple of 3X the mean number of students in the target year (*n* = 132).	
(v) Extending the benefit of multiple strategies to all students	Assumes benefit of PAS 1, PAS 5 and ISS 1, 2 and 3 are extended to all students (100 %) outside the target year (in Grades 7–10), with a reduced effect on daily minutes of MVPA compared to students in the target year.	As above, due to the nature of the intervention strategies (teacher training, school environment and broader school community links) the intervention impact would likely not be isolated to the evaluation cohort. For example, once PE teachers are trained on how to maximise MVPA in PE, these strategies would likely be applied to all classes at no additional cost. The same assumption applies for other strategies such as a school Physical activity policy, executive support, change agent, and use of resources. As such the cost of these strategies would not increase, however we have assumed there is potential for more students to benefit from a school implementing such strategies.
	The assumed effect size for the extension cohort was based on the results of the sensitivity analysis conducted within the efficacy trial (undertaken using imputation of missing data).	
Scenario analysis		
State wide roll out (current model)	Total cost of the intervention is based on the current implementation support model.	
	Assumes benefit to 100 % of students, with an effect size based on the results of the sensitivity analysis conducted within the efficacy trial (undertaken using imputation of missing data).	
	The number of students (n = 254,923) is based on a calculation from 487 NSW schools with Grades 7–10.	
State wide roll out- Alternate (real world) model	The total cost of the intervention is modified to reflect (a) an alternate model of school support - existing in-school teacher to support role out (1/2 day per week (0.5 FTE/ ½ day per week) and (b) a reduction in the equipment cost per school. Whilst the offer of an equipment pack was an attractive selling point for schools to consent to the intervention, evaluation of this specific strategy highlighted that schools within the intervention group were well stocked with equipment. As such, the provision equipment was not deemed an essential component of the trial. Based on this observation, the assumption that reducing the intervention costs by removing the provision of equipment, would not substantively alter the impact of the intervention.	
	Assumes benefit to 100 % of students, with an effect size based on the results of the sensitivity analysis conducted within the efficacy trial (undertaken using imputation of missing data).	
	The number of students (n = 254,923) is based on a calculation from 487 NSW schools with Grades 7–10.	

In addition, two scenario analyses, detailed in Table 1 were undertaken to explore the potential cost effectiveness of state-wide implementation of the intervention across NSW. There are 487 secondary schools catering for students in Grades 7 to 10 in NSW, with 254,923 students enrolled in these Grades. The first scenario used the current intervention implementation model within the target year across all applicable secondary schools in NSW. That is, those schools with Grades 7–10. Due to the logistical challenges of implementing interventions across large groups of schools and based on questions posed to principals of participating schools, the second scenario analysis used a real world solution whereby the implementation of school based physical activity practices is supported by an existing in-school teacher as an alternative to the school physical activity consultant employed in the efficacy trial. The potential model utilising an existing in-school teacher for providing guidance for schools was assumed for the intervention across Government and Catholic schools catering for students in Grades 7 to 10 across NSW (*n* = 487 secondary schools, catering for 254,923 students). The dissemination model included the costs of each school receiving relief funding for three periods per week for two years to support the implementation of the intervention within the school. This relief funding would allow an existing teacher within each secondary school to be released from classroom teaching to support the implementation within their school. This existing school teacher would be provided with teacher professional learning to enable them to embed the seven PA4E1 strategies within the school, using the same intervention implementation strategies used in PA4E1. Such a model was supported by principals of participating schools, who expressed a willingness to commit school resources for an in-school consultant for a period of 24 months. It was assumed that expansion of the intervention and changes to the support model would result in a reduced effectiveness compared to the primary trial outcome reported in the efficacy trial [60], and a reduced impact as outlined in the sensitivity analysis for students outside the target year.

## Results

### Schools

Five intervention schools (including 4 government and 1 catholic school of which 3 schools were located within the inner city and 2 were rural schools, with a mean of 129 Year 7 students) and five control schools ((including 4 government and 1 catholic school of which 3 schools were inner city schools and 2 were rural schools with a mean of 101 year 7students).

### Trial participants

The study included 1150 students in Grade 7 (645 intervention, 505 control) at baseline. At 24-month follow-up, 985 students wore an accelerometer with 441 students providing valid physical activity outcome data (three or more days of accelerometer data) and 985 students provided weight status outcome data. Table 2 outlines the characteristics of students in the sample.

**Table 2:** Student characteristics at baseline – students wearing an accelerometer (*n* = 1150)

Characteristic	Intervention group	Control group
Number/ Total Participants	645	505
Boys^a^	312	246
Girls^a^	333	258
3 vld days	530	435
Mean age (years)	12.0	12.0
Aboriginal and/ or Torres Strait Islander (%)	5.3 %	7.8 %
Height, (mean m)	157.1	156.8
Weight, (mean kg)	49.3	50.0
Student BMI Category, (%)	78.3 %	73.3 %
Underweight/ Healthy Weight		
Overweight/ Obese	21.7 %	24.7 %
Student activity level	33 %	33 %
Active (≥60 min MVPA/ day)		
Low active (<60 min MVPA/ day)	67 %	67 %
Accelerometer wear time	793.6	804.6
Mean minutes per day		

^a^Note - One (1) gender missing

### Trial outcomes

At 24-month follow-up, the adjusted mean difference in change in daily MVPA between groups was 7.0 min (95 % CI: 2.7, 11.4, *p* <0.002). Sensitivity analyses based on multiple imputation were consistent with the main analysis (6.0 min, 95 % CI: 0.6, 11.3, *p* < 0.031) [39]. The difference in change for BMI and BMI z-score was −0.28 (95 % CI = −0.49;–0.06, *p* = 0.01) and −0.08 (95 % CI = −0.14;–0.02, *p*–0.02) respectively, favouring the intervention group.

### Intervention costs

A total of 837 students were enrolled in Grade 7 at schools allocated to the intervention group of the study and were therefore included in the economic analysis. Table 3 shows the breakdown of the intervention costs against the various physical activity and implementation strategies. The total cost of the intervention was calculated to be $329,952 over 24 months. Unit costs of intervention components are displayed in Table 4. On the basis that schools allocated to either intervention or control would likely have the same baseline costs of implementing PE and sport, a zero cost was assumed for usual physical activity practices of schools randomised to the control arm, resulting in an intervention cost of $394 per student.

**Table 3:** Breakdown of costs across physical activity intervention and implementation strategies over two years

Physical activity intervention strategies (PAS)	Description & cost components	Total cost (24 m)	Total cost (24 m) per student	
1	Active PE lesson^a^	Teachers should maximise student activity in PE. 2 × pedometer based lessons per teacher each term used to build understanding of activity levels	$0	$0
2	Personal physical activity plans^a^	1 × personal student PA plan developed and reviewed each school term	$0	$0
3	Enhanced sports program^a^	Program X (10 week program) delivered to all students	$0	$0
4	Recess and lunchtime activities	Activities offered and equipment available at least twice per week	$10,526	$13
		Cost includes the opportunity cost of school staff time associated with monitoring and supervision of equipment use		
5	Supportive school physical activity policy ^a^	School PA policy modified or developed	$301	$0.36
		Cost includes the opportunity cost of school staff time to modify/ develop PA policy (four schools)		
6	Linking with parents	1 × hard copy parent newsletter per term focussed on physical activity. Newsletters also placed on school websites.	$4,933	$6
		Cost includes printing and materials		
7	Linking with the community	3–5 community links made – students made aware of community PA organisations	$8,285	$10
		Cost relates to community provider expos and includes showbag materials plus the opportunity cost of the preparation and face-face time of community sports representatives and school staff		
Implementation support strategies (ISS)				
1	In school consultant (change agent)	Attends school 1 day per week. Cost is salary for two years	$216,544	$259
2	School leadership & committee	Partnership agreement signed, School committee established. School executive membership represented on committee	$1,263	$1.51
		Cost includes the opportunity cost of school staff time associated with committee meeting attendance		
3	Staff development & training	Joint school professional development training	$28,340	$34
		Cost includes the opportunity cost of school staff time (teacher relief), external consultant services, travel and meal expenses and venue hire		
4	Resources	Physical Activity equipment pack (e.g. balls, hoops, ropes), recess and lunch equipment, class pedometer sets (5 per school), personal plans (templates and teacher instructions)	$59,370	$71
5	Prompts	Weekly email prompts to teachers from change agent	$389	$0.46
		Costs include printing and materials		
6	Performance feedback	Report delivered 1 x per term to Principal and head PE teacher	$0	$0
Total cost	$329,952	$394		

^a^Costs are accounted for in various implementation strategies

**Table 4:** Physical activity 4 Everyone intervention unit costs

Cost variable	Unit	Value
PE staff labour time	Rate/h	$60.15^a^
Volunteer personnel, labour time	Rate per hour	$33.18^b^
Printing	Cents per sheet	
Showbag contents	Cost per bag	$0.62^c^
Venue hire (including catering)	Cost per session	$482.133^c^
Conference fees	Cost per conference	$1805.00^c^
Travel expenses	Cost per person	$441.23^c^
Equipment packs (including incentives)	Per pack	$11,874.00^c^

Sources for cost prices

^a^Commission IR: Crown employees (Teachers in schools and related employees) salaries and conditions award 2014. In., vol. May; 2014

^b^Average weekly total cash earnings May 2014, ABS 6302.1

^c^Real cost price

### Incremental cost effectiveness ratios

Cost per additional minute of MVPA per day gained:

Based on the finding of a difference in change of 7.0 (95 % CI 2.68–11.36) minutes per student per day of MVPA for students in the intervention versus control groups [39], the intervention cost of $394 per student divided by 7.0 resulted in an incremental cost effectiveness ratio of $56 [95 % CI $35–$147] per additional minute of MVPA per day (Tables 3 and 5).

**Table 5:** Mean costs per participant, mean difference in change and ICER’s presented for physical activity (MVPA and MET minutes) and weight status (BMI unit avoided and per 0.1 unit (10 %) reduction in BMI z-score

	Cost per enrolled student in five intervention schools over 24 months	Mean difference in change between Intervention and Control groups at 24 month follow-up (95 % CI)	ICER (95 % CI)
Mean minutes MVPA/ Day	$394	7.0 (2.7–11.3)	$56 ($35–$147)^a^
MET hours gained per person/ day		0.5 (0.2–0.9)	$749 ($463–$1,961)^b^
BMI		0.3 (0.1–0.5)	$1408 ($788–$6,570)^c^
BMI z-score		0.1 (0.0–0.1)	$563 (282–3,942)^d^

^a^ cost per minutes of MVPA gained

^b^cost per MET hour gained

^c^cost to avoid a gain in1 BMI unit

^d^cost per 0.1 (10 %) unit reduction in BMI z-score

Cost per MET hour gained per person per day:

When mean minutes MVPA per day were converted to MET hours gained, the PA4E1 intervention resulted in 0.5 [95 % CI 0.2–0.9] MET hours gained per person/ day, and a cost of effectiveness ratio of $1 ($0.6–$2.7 per MET hour gained (Table 3).

Cost per BMI unit avoided:

Based on a finding of a difference in change of −0.28 BMI units per student in the intervention group versus the control group [40], the intervention cost of $394 per student divided by −0.28 resulted in an incremental cost effectiveness ratio of $1,408 [95 % CI $788–$6,570] per BMI unit avoided (Table 3).

Cost per reduction in BMI z-score:

Similarly, the intervention cost of $394 per student divided by the difference in BMI z-score of −0.07 [40], resulted in an incremental cost effectiveness ratio of $5,632 per 1.0 unit BMI z-score reduction or $563 per 10 % reduction in BMI z-score [95 % CI $282–$3,942] (Table 3).

Students included all students enrolled in Grade 7 at intervention commencement.

### Sensitivity analysis

**Fig. 2:**
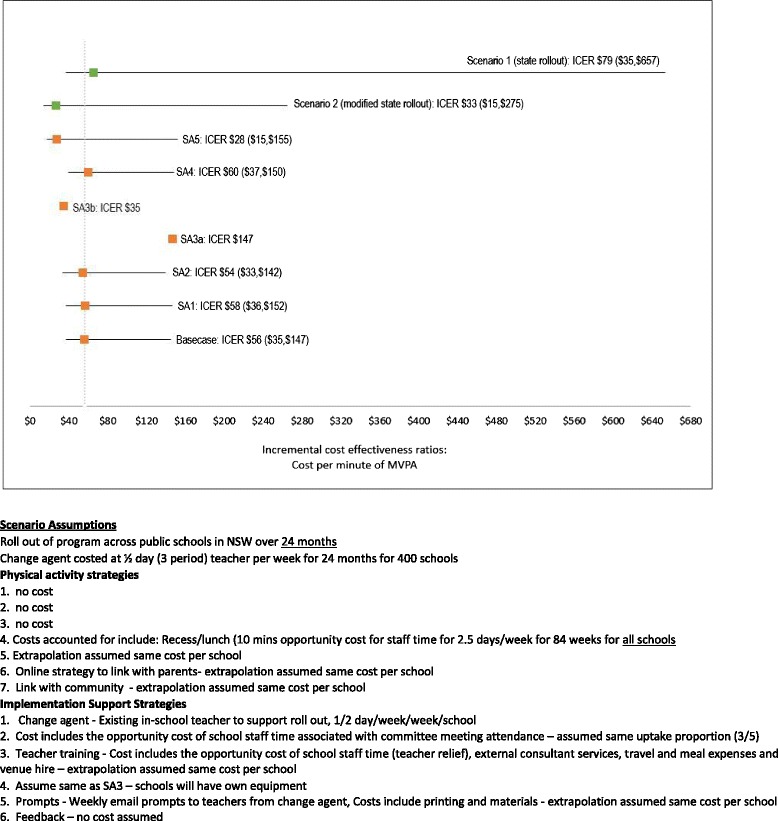
Sensitivity and scenario analyses for PA4E1 intervention

Figure 2 outlines the outcomes from sensitivity testing. Tests (i) and (ii) plausible variation in the cost of the intervention by varying the assumed opportunity cost of school staff participation in PAS 4 & 5 and ISS 2 resulted in ICERs of $57 ($35, $149) and $54 ($33, $142) respectively. Test (iii) variation in the magnitude of the estimated effect size between the lower and upper confidence interval in minutes of MVPA per day resulted in point estimate ICERs of $35 and $147 respectively. Tests (iv) and (v) extending the intervention benefit outside the target grade resulted in ICERs of $60 ($37, $150) and $28 ($15, $154) respectively.

### Scenario analysis

The costs to disseminate the intervention across secondary schools in NSW using the existing model were $66 ($35–$656) per additional minute of MVPA. The cost of disseminating the intervention across NSW, through a real world model provision of teacher relief funding for half a day per week over 24 months to allow an existing in teacher to lead the implementation of the program at school (estimated to cost $10,100 per school over 24 months) resulted in a cost per minute of MVPA of $27 ($14–$267) (Fig. 2).

## Discussion

This study assessed the cost and cost effectiveness of a multi-component school-based intervention (*Physical Activity 4 Every1*) that aimed to reduce the decline in physical activity among secondary school students. The cost of the intervention was $329,952 over a 24-month period, resulting in the intervention being delivered at a cost of $394 per student. In terms of physical activity, the ICER was $56 per minute of MVPA gained and $1 per MET hour gained per person. From a weight perspective, the ICER’s were $1,408 per BMI unit avoided and $563 per 10 % reduction in BMI z-score. These findings suggest that implementation of the intervention by schools in disadvantaged areas has the potential to make a cost-effective contribution to reducing the decline in physical activity during adolescence and the health-related burden associated with physical inactivity and overweight and obesity.

This is one of the few cost effectiveness studies of school-based physical activity interventions targeting adolescents, and to the authors’ knowledge, the first based on an objective measure of physical activity, and the first cost effectiveness study of a school-based physical activity intervention targeting disadvantaged adolescents. While, the PA4E1 intervention demonstrated a consistent effect in terms of MET hours gained per person/day compared to a meta-analysis of the cost effectiveness of school-based physical activity interventions (0.50 compared to 0.48 MET hours gained) [31], the cost effectiveness profiles of the studies are not as easily compared. The cost-effectiveness result from the PA4E1 intervention of $1 per MET hour gained is at the upper end of the spectrum of reported cost-effectiveness ratios of the studies included in the reviews ($0.06–$0.8/MET hr). However, as discussed above, the reported costs and therefore cost-effectiveness of the studies included in the meta-analyses were derived from either published cost analyses or imputed by the review authors and therefore may not accurately reflect the profiles of the interventions. Since the current analysis did not extend to including any potential cost-offsets associated with increased physical activity, the cost-effectiveness of the intervention should still be considered favourable.

From a weight perspective, the intervention costs per child calculated in PA4E1 are similar to a school- and community-based childhood obesity intervention (implementing both nutrition and physical activity strategies) known as *Be Active Eat Well*, which was also implemented in Australia targeting children aged 5–12 years [29]. This study reported a cost per child of $344AUD, and resulted in a similar effect on BMI (0.28 BMI Units), but a greater impact on BMI Z-score [29], potentially due to the younger age of the students targeted by the intervention [22]. Similarly, the APPLES childhood obesity prevention study conducted in New Zealand targeted children aged 5–12 years and reported higher intervention cost per child of NZD $1,281 (equivalent to $1202.7AUD), and an incremental cost-effectiveness ratio (ICER) of NZD $664–$1708 per kg of weight-gain prevented [35].

The cost per student in the PA4E1 study were comparable to other school-based physical activity interventions and multi-component school-based obesity prevention interventions with a physical education component that have reported to be cost effective [29, 33, 61]. This is in spite of PA4E1 targeting adolescents, in which systematic reviews show smaller effects in adolescents compared to elementary aged children. As a result, the PA4E1 study seems a cost effective option for improving the physical activity and weight status of adolescents within a higher risk population group [29, 33]. In most cases, the cost effectiveness ratios are conservative in nature due to the intervention effect being limited to the target group only. Sensitivity analyses revealed lower costs per students when the benefits were extended beyond the target group to others students in the school, or if equipment provided was reduced.

Based on conservative estimates of benefit (applied to the target year only), this study demonstrates that PA4E1 is a cost effective intervention for maintaining adolescent physical activity levels and impacting favourably on weight status. The sensitivity analyses provide insight into impact of the intervention if the health benefits were applied to students across the school more broadly, with the majority of these analyse demonstrating a greater cost effectiveness and a reduced intervention cost per student. When the assumptions of the intervention are manipulated as demonstrated in the scenario analyses, by reducing the cost of equipment and extending the benefit of the MVPA outcome (at a reduced level) beyond the target year, the intervention remains cost effective. The provision of an in-school physical activity consultant for one day per week was the largest cost relating to the efficacy trial (66 % of the total intervention cost). Whilst the provision of an in-school physical activity consultant was necessary under efficacy trial conditions in order to evaluate the effect of the combination of intervention strategies, the feasibility of providing a part-time consultant within schools across large geographic regions and the cost of such a model of support presents challenges in upscaling the intervention. The dissemination of an effective intervention across the community requires the use of implementation strategies which better mirror real world practice. A dissemination model that utilises an existing in-school teacher to embed desired practices has been shown to successfully impact on student physical activity levels, and our results indicate such a model is more cost effective at scale [45, 62]. However, to the authors’ knowledge, the cost effectiveness of these studies has not been reported. Whilst PA4E1 appears to be a cost effective intervention, dissemination is needed if its health benefits are to be realised. Based on a model to a disseminate an effective intervention under real world conditions, a scenario analysis indicated the potential of a state-wide roll-out of the PA4E1 program, resulting in a cost per student which was substantially reduced compared to the costs of the randomised controlled trial. As the intervention is effective, prioritising higher risk schools such as those located in socio-economically disadvantaged areas may provide a rationale for prioritisation.

### Strengths and limitations

This study has a number of strengths and limitations that should be considered within the broader context of the economic evaluations and disease prevention. The strengths include: firstly the data informing the analysis is based on results from a randomized controlled trial using [16, 35, 39, 63] objectively measured physical activity using accelerometry. Secondly, the costs associated with the intervention were collected prospectively thus improving accuracy by eliminating recall bias [64]. Thirdly, this study reported the ICER from a number of perspectives, both physical activity and weight status. This enables comparison across studies, particularly physical activity studies in which a broad range of outcomes have been used in the past and therefore limit the usefulness of such studies. In our case, the conversion of the physical activity outcome to METS, and cost per MET minute gained enable useful comparison with the limited number of published physical activity cost effectiveness studies [31].

The study also has limitations that should be noted. The translation of the intermediate outcomes captured by the study into final outcomes, such as DALYs, expedient for economic evaluations was beyond the scope of this analysis. This type of modelling has previously been conducted on interventions that aimed to prevent overweight and obesity in children and adolescents, and as a result may provide policy makers with additional useful data to make informed policy decisions [29]. These studies model the broad societal level cost effectiveness, and should potentially be considered for this intervention in the future.

The sensitivity and scenario analysis are both hypothetical. Whilst based on empirical data from the evaluation of the intervention they may overestimate (or underestimate) the impact of changing the intervention component on the intervention costs. The scenario analysis tests only one set of possible assumptions, and whilst based on empirical data collected via a sensitivity analysis conducted within the efficacy trial and formative research of schools participating in the intervention, the scenario is hypothetical. Additionally, this analysis is constrained by the time horizon of the intervention. Whilst the intervention appears to be cost effective and able to obtain health benefits for both physical activity and weight status for a relatively low cost, the sustainability of these behaviours remains unknown. Lai and colleagues [65] have indicated the physical activity of similar school-based intervention can be sustained, however, the likelihood that the positive change achieved through the PA4E1 intervention can be maintained is currently unknown. Future research on the sustainability of PA4E1 is warranted in addition to research evaluating the impact of using an alternative model to support large scale implementation. This would in turn inform the extrapolation of these cost effectiveness results.

## Conclusion

The PA4E1 intervention had a statistically significant intervention effect on physical activity and weight gain which can be achieved for a relatively low monetary cost of $394AUD per student over a 24-month period. This investment is promising for public health policy, particularly as the intervention was delivered in school communities located in disadvantaged communities where both physical inactivity and overweight and obesity are likely to be more prevalent, therefore likely to result in a greater future burden of disease. Further research is required to determine the impact of the intervention if implemented on a routine basis throughout the period of secondary schooling.

## Abbreviations

BMI: Body mass index
ICER: Incremental cost effectiveness ratios
ISS: Implementation support strategy
MVPA: Moderate-to-vigorous physical activity
NSW: New South Wales
PA4E1: Physical activity 4 everyone
PAS: Physical activity strategy
PE: Physical education
